# Valproic Acid and Fatalities in Children: A Review of Individual Case Safety Reports in VigiBase

**DOI:** 10.1371/journal.pone.0108970

**Published:** 2014-10-10

**Authors:** Kristina Star, I. Ralph Edwards, Imti Choonara

**Affiliations:** 1 Uppsala Monitoring Centre, WHO Collaborating Centre for International Drug Monitoring, Uppsala, Sweden; 2 Academic Division of Child Health, University of Nottingham, Derbyshire Children's Hospital, Nottingham, United Kingdom; Louisiana State University Health Sciences center, United States of America

## Abstract

**Introduction:**

Valproic acid is an effective first line drug for the treatment of epilepsy. Hepatotoxicity is a rare and potentially fatal adverse reaction for this medicine.

**Objective:**

Firstly to characterise valproic acid reports on children with fatal outcome and secondly to determine reporting over time of hepatotoxicity with fatal outcome.

**Methods:**

Individual case safety reports (ICSRs) for children ≤17 years with valproic acid and fatal outcome were retrieved from the WHO Global ICSR database, VigiBase, in June 2013. Reports were classified into hepatotoxic reactions or other reactions. Shrinkage observed-to-expected ratios were used to explore the relative reporting trend over time and for patient age. The frequency of polytherapy, i.e. reports with more than one antiepileptic medicine, was investigated.

**Results:**

There have been 268 ICSRs with valproic acid and fatal outcome in children, reported from 25 countries since 1977. A total of 156 fatalities were reported with hepatotoxicity, which has been continuously and disproportionally reported over time. There were 31 fatalities with pancreatitis. Other frequently reported events were coma/encephalopathy, seizures, respiratory disorders and coagulopathy. Hepatotoxicity was disproportionally and most commonly reported in children aged 6 years and under (104/156 reports) but affected children of all ages. Polytherapy was significantly more frequently reported for valproic acid with fatal outcome (58%) compared with non-fatal outcome (34%).

**Conclusion:**

Hepatotoxicity remains a considerable problem. The risk appears to be greatest in young children (6 years and below) but can occur at any age. Polytherapy is commonly reported and seems to be a risk factor for hepatotoxicity, pancreatitis and other serious adverse drug reactions with valproic acid.

## Introduction

Valproic acid is a widely used antiepileptic drug (AED) that is highly effective both in adults and children. It is one of the first line drugs for the treatment of epilepsy. It was first used in Europe in 1968 and in the USA in 1978 [1]. The most frequent adverse drug reactions (ADRs) of valproic acid include somnolence, weight gain, fatigue and headache [2]. The most serious ADRs include hepatotoxicity and pancreatitis, both of which can be fatal.

The first case reports of hepatotoxicity following the use of valproic acid were in the late seventies [3], [4]. In 1987 a comprehensive review of 37 cases of fatal hepatotoxicity in the USA occurring in patients receiving valproic acid between 1978 and 1984 was carried out [5]. This comprehensive review by Dreifuss identified three major risk factors. The risk was greatest in children under the age of three years, in patients on polytherapy and those with signs of developmental delay [5]. A year later, Scheffner described the deaths of 16 children in Germany [6]. Eleven of these children were on polytherapy and 11 had developmental delay. Only two of the 16 children, however, were under the age of three years. In 1989 Dreifuss again reviewed the US data and described a significant reduction in mortality associated with valproic acid in the USA and suggested that more rational prescribing in terms of using valproic acid as monotherapy had resulted in decreased mortality [7]. Other rare ADRs of valproic acid that may result in death include pancreatitis [8].

In a British review of suspected ADRs and fatalities in children, valproic acid was the AED most frequently reported with a fatality [9]. We were therefore interested in characterising suspected ADRs with fatal outcome for valproic acid reported worldwide and to determine whether hepatotoxicity with fatal outcome was still reported as a considerable problem following the guidance suggested by Dreifuss.

## Methods

The WHO Collaborating Centre for International Drug Monitoring in Uppsala, Sweden, i.e. the Uppsala Monitoring Centre (UMC), receives individual case safety reports (ICSRs) of suspected ADRs from national pharmacovigilance centres around the world. The reports are stored in the WHO Global ICSR database, VigiBase [10]. The database is an appropriate source to retrieve information on rare ADRs particularly in subpopulations such as in children because of its worldwide coverage [11]. More than 8 million ICSRs from more than 100 countries had been compiled in VigiBase up to June 2013.

ICSRs for children (age 1 month to 17 years) recorded with valproic acid (suspected or interacting), according to the preferred base in the WHO Drug Dictionary Enhanced, were extracted from VigiBase containing data up to June 2013. Fatalities were defined as reports with adverse reactions recorded with outcome ‘died’, or reports coded with a term indicating death (e.g. 'Sudden death' or ‘Death’), or with the seriousness criteria classified as ‘death’, or recorded with a post-mortem code.

Suspected duplicate reports were excluded by using an automated screening method applied to VigiBase data and previously described in detail [12]. A manual screening for additional duplicates was subsequently performed. However, duplicate reports could still remain, especially when containing very little data. Reports specified as being sent by lawyers or recorded as originating from published literature cases were excluded from the analysis. Reports originating from the US are not coded to be literature reports or not, therefore these types of reports could still remain in the data retrieved for this study. The restriction of using reports 1 month of age and above was applied to reduce the number of reports relating to maternal exposure. A manual review to remove reports with congenital anomalies resulting from maternal exposure to valproic acid was also performed. Reports with reactions that were most unlikely to be the cause of death were also excluded.

Reports were grouped into being related to hepatotoxicity or not. Several suspected ADRs can be listed on a report and when any of the reported terms was subordinated to the Medical Dictionary for Regulatory Activities (MedDRA) ‘Hepatic and hepatobiliary disorders’ (High-level group term), ‘Liver function analyses’ (High-level term), ‘Coma hepatic’, ‘Hepatic encephalopathy’ (Preferred terms), the report was classified as related to hepatotoxicity. A manual review of all terms for each report was also performed resulting in three additional reports with hyperammonaemia and encephalopathy to be classified to the group of hepatotoxic reports.

The case series considered in this study with valproic acid and fatal outcome in the child age group were:

- All fatal reports with and without hepatotoxic events.
- Fatal reports with hepatotoxic events (as previously defined).
- Fatal reports without hepatotoxic events.

To define the timing of an event in this study, the onset date of the event was used if completed on the report. If this was not available the date was estimated, using firstly the suspected drug stop date, or otherwise the date of when the national centre received the report, or lastly the date of first VigiBase entry. Reporting by time of the event (or estimated as described previously) was investigated. The first time period encompassed the period 1977–1990 when hepatotoxicity was first recognised and three key reviews describing risk factors of hepatotoxicity were published [5]–[7]. The second time period was 1991 up to June 2013.

Relative reporting trends were graphically displayed by using shrinkage observed-to-expected ratios on a logarithmic scale [13], [14]. The observed value consisted of the number of reports within each case series as defined above and grouped by year of age or time period. The expected number was computed based on the total number of reports for valproic acid, the total number of the event; i.e. fatalities with hepatotoxicity or fatalities without hepatotoxicity; and the total number of reports for the children (any drug or event), by year of age or time period. The measure is referred to as the Information Component (IC), which is given with its 95% credibility interval (IC025), where the lower positive value is used to highlight disproportional reporting. The observed and expected numbers are also included in the graphs. Non-cumulative reporting for 5-year time periods and for each yearly patient age is displayed.

Reports with more than one reported AED (i.e., reports listing AEDs in addition to valproic acid) were considered cases of polytherapy in this study. The co-reported drugs could be assigned by the original reporter to be suspect, interacting or concomitant. Reporting of polytherapy for the three case series with fatal outcome were compared with corresponding groups of non-fatal reports for valproic acid using log shrinkage odds ratios.

### Ethics statement

De-identified individual case safety reports have been routinely collected as a public health service internationally since 1968, through the WHO Programme for International Drug Monitoring. The protection of the identity of the patient and the reporter has been routine from the outset.

## Results

After exclusion of literature reports (n = 42), reports concerning lawyers (n = 1), suspected duplicates (n = 63), maternal exposure (n = 8) and non-fatal events (n = 5), 268 reports remained and were analysed. Reports had been received from 25 countries worldwide from 1977 until the end of 2012. Ages ranged from 2 months to 17 years, and 54% of the reports with a recorded patient sex concerned boys. In 156 reports the fatalities were recorded with hepatotoxicity, of which hepatic failure, hepatic necrosis, abnormal hepatic function and hepatocellular injury were most frequently reported. The median number of days from start of valproic acid to onset of reaction (time-to-onset) for the group with hepatotoxic reactions was 66 days (IQR: 38.75–121.5) and for the group without hepatotoxic reactions specified, the median time-to-onset was 130 days (IQR: 54–478). The median time-to-onset is based on reports where dates needed for the calculation had been recorded.

**Figure 1:**
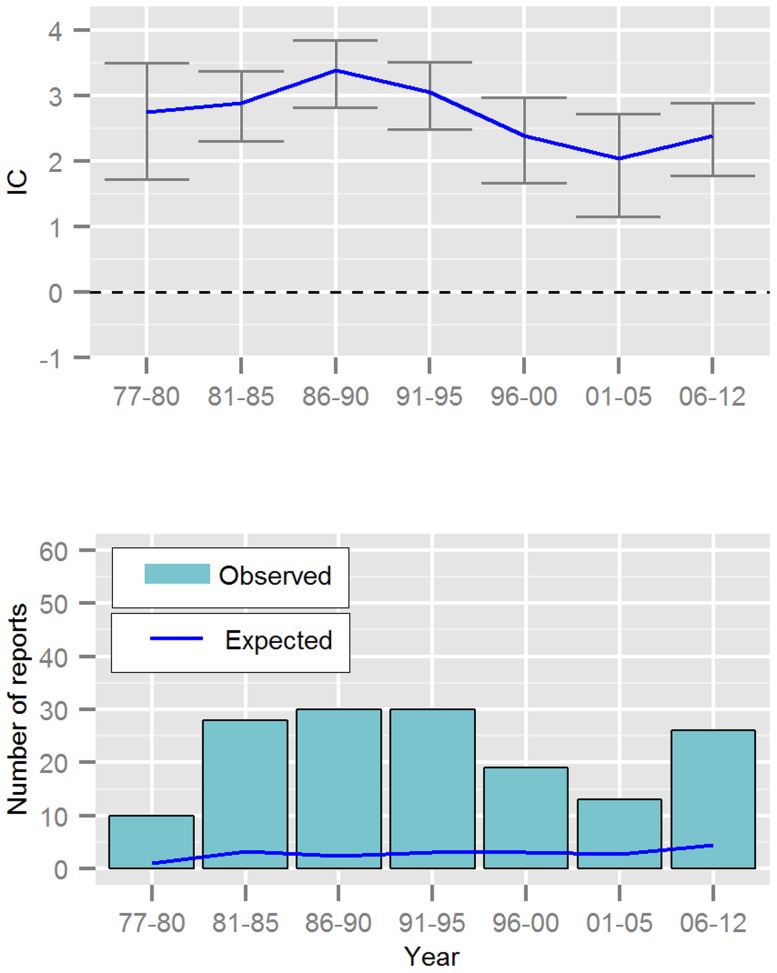
Reporting over time for valproic acid and fatalities *with* hepatotoxicity; displaying the Information Component (IC) and its 95% credibility interval, observed and expected number of reports from VigiBase. The timing of the report is based on the estimated onset date of the event.

**Figure 2:**
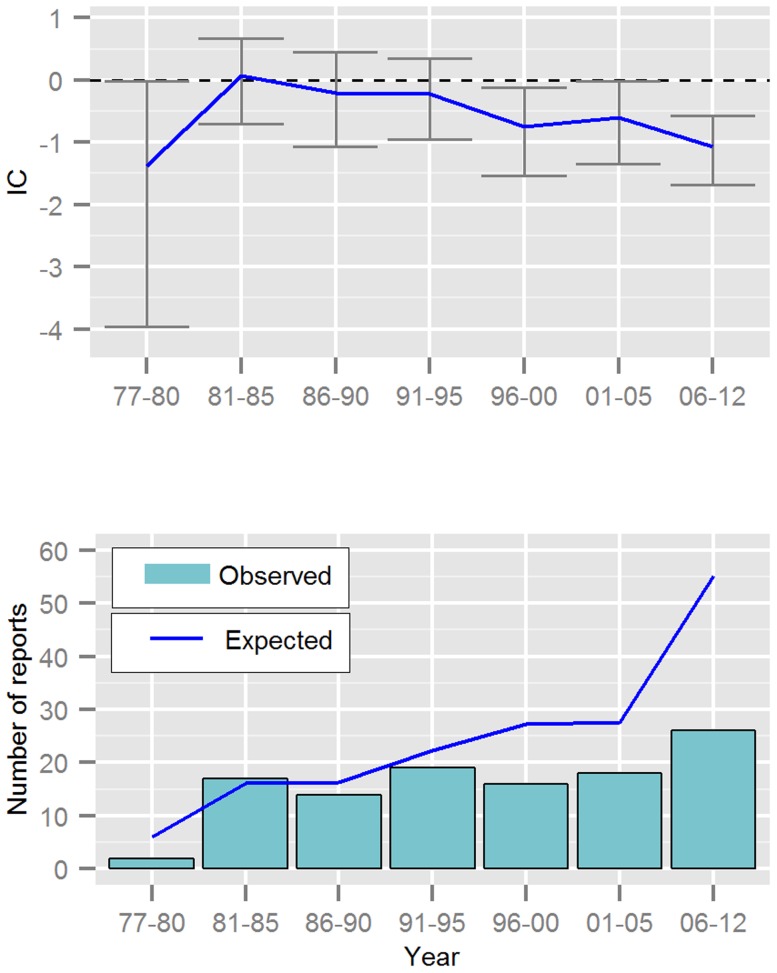
Reporting over time for valproic acid and fatalities *without* hepatotoxicity; displaying the Information Component (IC) and its 95% credibility interval, observed and expected number of reports from VigiBase. The timing of the report is based on the estimated onset date of the event.

The number of fatalities each year has ranged from one to 24 reports. The greatest number of reported fatalities occurred in 1983 (16 cases of hepatotoxicity and eight others). The mean number of fatalities reported per year was 7.4 reports (Table 1) with little change before and after 1990. Between 1977 and 1990, 68 fatalities with hepatotoxicity were reported. There were 88 fatalities reported with hepatotoxicity in the second time period (1991–2012). However, the overall mean reporting of valproic acid had increased from 122 per year to 230 after 1990 (Table 1). Fatalities with hepatotoxicity have been disproportionally reported throughout the complete time period (Figure 1). Non-hepatotoxic fatalities have been evenly reported over time, although less frequently than what was expected in VigiBase (Figure 2).

**Table 1:** Overall reporting of valproic acid with fatal outcome in children before and after 1990.

Year of reaction	No. reports with valproic acid and fatal outcome	Mean No. reports with valproic acid and fatal outcome per year	No. reports with valproic acid	Mean No. reports with valproic acid per year
1977–1990	101	7.2	1702	122
1991–2012	167	7.6	5058	230
**1977–2012**	**268**	**7.4**	**6760**	**188**

**Figure 3:**
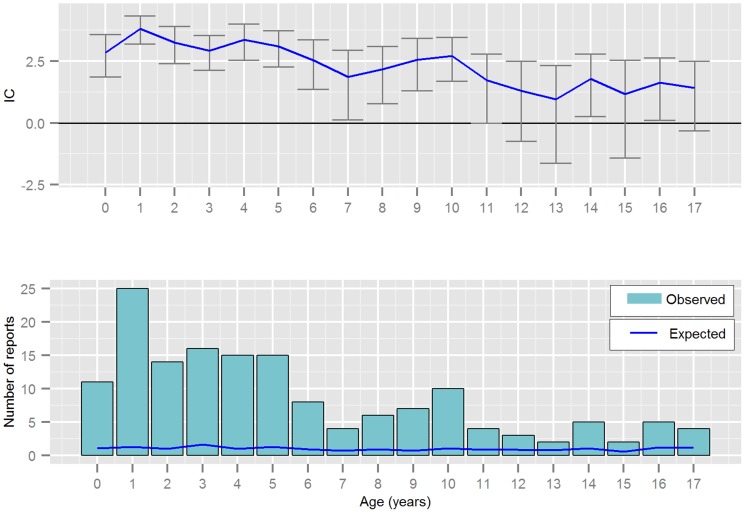
Reporting by patient age in years for valproic acid and fatalities *with* hepatotoxicity; displaying the Information Component (IC) and its 95% credibility interval, observed and expected number of reports from VigiBase.

**Figure 4:**
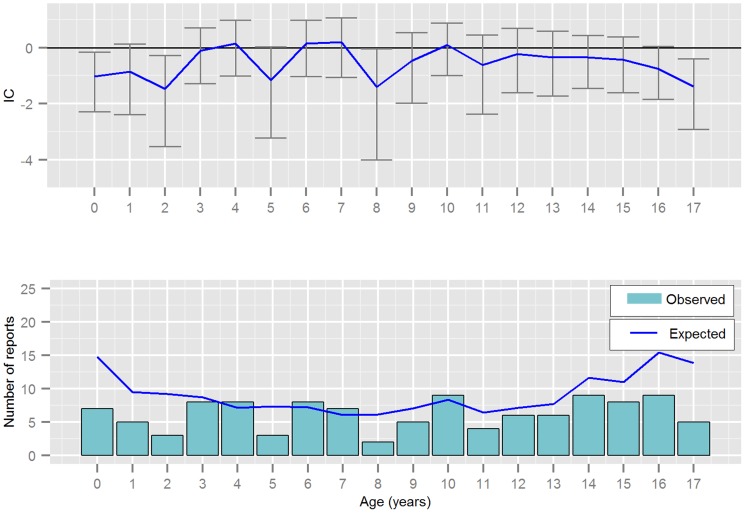
Reporting by patient age in years for valproic acid and fatalities *without* hepatotoxicity; displaying the Information Component (IC) and its 95% credibility interval, observed and expected number of reports from VigiBase.

Hepatotoxicity was disproportionally reported for almost all ages included in this study (Figure 3), whilst reports without hepatotoxicity were not (Figure 4). Hepatotoxicity was most commonly reported in children aged 6 years and under (104/156 reports). The median ages and frequency of AED polytherapy before and after 1990 are illustrated in Table 2. The table includes both fatalities with hepatotoxicity and those without hepatotoxicity. The relationship between polytherapy and age is shown more completely in Table 3.

**Table 2:** Overall reporting of valproic acid and fatal outcome reports in children with and without hepatotoxicity before and after 1990.

Type of reaction	Year of reaction	No. reports	Mean No. reports per year	Median age (years)	IQR age (years)	Polytherapy* No. reports (%)
Hepatotoxicity	1977–1990	68	4.9	4	2–8	45 (66)
	1991–2012	88	4.0	5	2–9.25	48 (55)
Not hepatotoxicity	1977–1990	33	2.4	6	2–7	20 (61)
	1991–2012	79	3.6	11	6.5–14.5	42 (53)

*Reports recorded with more than one suspected, interacting or concomitant antiepileptic medicine.

**Table 3:** Age groups and polytherapy* for valproic acid and fatal outcome reports, with and without hepatoxicity.

	Hepatotoxicity		Not hepatotoxicity	
Age groups (years)	No. reports	Polytherapy* No. reports (%)	No. reports	Polytherapy* No. reports (%)
0 to 2	50	34 (68)	15	7 (47)
3 to 6	54	34 (63)	27	17 (63)
7 to 11	31	14 (45)	27	17 (63)
12 to 17	21	11 (52)	43	21 (49)

*Reports recorded with more than one suspected, interacting or concomitant antiepileptic drug.

In 155 (58%) reports, one or more AEDs were reported alongside valproic acid. A total of 28 different AEDs were reported and the ten most frequently reported AEDs in the hepatotoxic and non-hepatotoxic groups are listed in Table 4. Phenobarbital, phenytoin and carbamazepine were the most frequently co-reported AEDs. Polytherapy was significantly more frequently reported for the three case series with fatalities contrasted with corresponding non-fatal group of reports (Table 5).

**Table 4:** The ten most frequently co-reported antiepileptic drugs with valproic acid and fatal outcome in children.*

Drug	Total No. reports	No. reports with hepatotoxicity	No. reports *without* hepatotoxicity
Phenobarbital	54	44	10
Phenytoin	50	38	12
Carbamazepine	45	28	17
Clonazepam	26	17	9
Diazepam	19	7	12
Lamotrigine	11	6	5
Clobazam	7	3	4
Lorazepam	7	6	1
Topiramate	6	3	3
Levetiracetam	5	2	3

*Numbers do not add up, since more than one antiepileptic drug (AED) could be recorded on one report. AEDs could have been recorded as suspected, interacting or concomitant medicines on the reports.

**Table 5:** Valproic acid reports with polytherapy* and fatal outcome were contrasted with non-fatal outcome reports in children, grouped by reports with or without hepatotoxicity, or with any event.

	Valproic acid and fatal outcome		Valproic acid *without* fatal outcome		Log shrinkage odds ratios (OR)**	
	No. reports	Polytherapy* No. reports (%)	No. reports	Polytherapy* No. reports (%)	OR	OR005
Hepatotoxicity	156	93 (60)	695	323 (46)	0.76	0.35
Not hepatotoxicity	112	62 (55)	5755	1892 (33)	1.32	0.81
Any event	268	155 (58)	6450	2215 (34)	1.38	1.07

*Reports recorded with more than one suspected, interacting or concomitant antiepileptic drug.

**The lower level of the 99% credibility interval for the log shrinkage odds ratio (OR005) was considered significant when above zero.

Many of the cases had more than one suspected ADR listed. Among the 268 reports, the most frequently reported events (apart from hepatotoxicity) were coma/encephalopathy, convulsion, pancreatitis, respiratory disorders, coagulopathy, thrombocytopenia and cardiac arrest/ventricular arrhythmias (Table 6). The second largest group consisted of children who were in a coma/encephalopathy, of which 35 of the 49 reports were co-reported with a hepatotoxic reaction. A large group of reports involved children who died in status epilepticus or following a seizure. It is unlikely that these were ADRs as such, but rather that the drug was ineffective. A number of events, not usually associated with valproic acid were reported: metabolic acidosis, disseminated intravascular coagulation, hyperammonaemia, apnoea, toxic epidermal necrolysis (TEN) and aplastic anaemia. Apart from the last two, the others seemed to be events complicating seriously ill patients.

**Table 6:** The most frequently reported adverse reactions for valproic acid with fatal outcome in children.

Adverse reaction	No. reports			Median age (years)	Polytherapy** No. reports (%)
	Total	Hep*	Non-Hep*		
Hepatotoxicity	156	156	0	4	93 (60)
Coma states/Disturbances in consciousness/Encephalopathies	49	35	14	5	31 (63)
Seizures	42	28	14	7.5	27 (64)
Pancreatitis	31	11	20	9	16 (52)
Respiratory disorders (apnoea, respiratory depression/failure/arrest)	27	10	17	7	17 (63)
Coagulopathy/Disseminated intravascular coagulation	25	19	6	7	19 (76)
Thrombocytopenias	21	10	11	4	14 (67)
Cardiac arrest/Ventricular arrhythmias	18	5	13	11.5	8 (44)
Infections (sepsis, pneumonia)	18	10	8	8	14 (78)
Anaemias (incl. aplastic) and marrow depression	13	3	10	5	10 (77)
Renal disorders (excl nephropathies)	12	7	5	7	9 (75)
Overdose (intentional/accidental/suicide)	11	0	11	12	2 (18)
Acidosis/Metabolic acidosis/Lactic acidosis	10	6	4	6	7 (70)
Brain oedema/Increased intracranial pressure	10	6	4	7	4 (40)
Haemorrhages (incl. cerebral)	7	3	4	7	3 (43)
Gastrointestinal haemorrhages	6	4	2	8	3 (50)
Toxic epidermal necrolysis/Stevens-Johnson syndrome	6	0	6	10	5 (83)
Hyperammonaemia	4	4	0	3.5	2 (50)

Numbers do not add up, since one report can be recorded with more than one reaction.

*‘Hep’ are reports with hepatotoxicity events recorded and ‘Non-Hep’ reports are without hepatotoxicity events recorded.

**Reports recorded with more than one suspected, interacting or concomitant antiepileptic drug.

## Discussion

Valproic acid with fatal outcome and hepatotoxicity has continuously been reported disproportionally since 1977. Polytherapy was significantly more frequently reported in fatalities than for non-fatalities and appears to remain as a considerable risk factor for serious ADRs, including hepatotoxicity. The younger age groups reported with hepatotoxicity had the highest proportions of co-reported AEDs, suggesting that these ages are particularly vulnerable to polytherapy. This is in keeping with the previous reviews of fatal hepatotoxicity in association with valproic acid [5], [6]. In the 70s, many patients with epilepsy (adults and children) received polytherapy [15]. A prospective study in adults in the late 1970s showed that monotherapy was effective for epilepsy and also reduced the risk of ADRs [15].

Although the greatest number of fatal cases of hepatotoxicity occurred in children aged between 1 and 2 years (Figure 1), there were a considerable number of cases in children of all ages, especially in children aged six years and below. It has been suggested that young children (less than 7.5 years) have an increased risk of hepatotoxicity due to their abnormal metabolism of valproic acid [16]. Clinicians need to be aware that the risk of fatal hepatotoxicity appears to be greatest in young children (six years and below) but that it can occur at any age. This corresponds with two recent reviews of German fatal and non-fatal cases where the risk of valproic acid associated hepatotoxicity was not restricted to infants [17], [18].

Unfortunately, for many reports there was insufficient information to be certain of the full nature of the suspected ADR (coma, convulsion and cardiac/respiratory arrest). Fatalities reported with pancreatitis occurred in 31 reports. Pancreatitis was first reported in 1979 and subsequently there have been several reviews and case series [19], [20]. One study, in a single hospital, identified 22 children with pancreatitis (diagnosed by using strict criteria) over a 10-year period [21]. This suggests that many cases of valproic toxicity resulting in pancreatitis are not reported. Polytherapy was not identified as a risk factor in any of the previous case series or reviews. The majority of patients with pancreatitis in a review of the literature, however, were receiving polytherapy [19]. In relation to reports in VigiBase, polytherapy could be a risk factor for pancreatitis.

Coagulopathies secondary to valproic acid are thought to occur in up to 4% of children [22]. Thrombocytopenia is thought to occur in between 5 and 40% of children receiving valproate [23], [24]. In the vast majority of cases, the coagulopathies are minor and result in no clinical problems. Factors associated with increased clinical severity of the coagulopathies are undetermined and needs further investigation.

Both aplastic anaemia and TEN are extremely rare ADRs in patients receiving AEDs. Aplastic anaemia has been reported in adults receiving valproic acid [25]. It has only previously been reported in paediatric patients in isolated case reports [23], [26], [27]. Similarly, Stevens-Johnson syndrome (SJS) and TEN have predominantly been reported in adults [28] with only isolated cases described in children on valproic acid [29]. The six cases with SJS or TEN in our study originated from six separate countries and were co-reported with phenobarbital (n = 2); carbamazepine; lamotrigine plus acetazolamide; paroxetine; and carbamazepine plus phenytoin. In an overall review of VigiBase reports (any age and with any outcome), a disproportionally higher reporting frequency was noted for children compared with adults for valproic acid and aplastic anaemia. A higher number of reports for aplastic anaemia in children with fatal outcome were also noted (5/8 cases were children).

This study presents the *reporting* patterns with its caveat for valproic acid and children with fatal outcome, see further Appendix S1 [30]. Reports in VigiBase come from a variety of sources and the likelihood that the reported suspected adverse reaction is drug-related is not the same in all cases. VigiBase reports are stored in structured format with basic information about the patient, the drug and adverse reaction. Narratives describing the case in more detail are rarely available electronically. In this study we only used the VigiBase data available in structured format, therefore case details to confirm a diagnosis or to assess the likelihood of a causal relationship between a drug and event was limited.

Several of the most commonly co-reported AEDs in this study can in themselves be associated with liver damage. As a consequence, there is uncertainty as to whether it was valproic acid or the co-reported drug that induced the reaction. To reduce this uncertainty, our report extraction generated only reports where valproic acid had been considered the suspected drug by the reporter.

Another limitation of the data is that co-reported AEDs may have been omitted on the report, which could have resulted in an underestimation of polytherapy in this study. However, we also made an assumption that the recorded AEDs with missing treatment dates had been used concomitantly with valproic acid so we may have overestimated the frequency of polytherapy.

There may have been a reporting bias towards reporting more young children with hepatotoxicity as their susceptibility to hepatotoxicity had been reported previously [5]. The problem of valproic acid and hepatotoxicity were introduced in the scientific literature towards the end of 1980. This might have introduced a bias of increased reporting around this time. The decreased relative reporting after 1996 might therefore be an artefact explained by unusually higher reporting before this time period. The decreased relative reporting frequency could also reflect that the problem is occurring less because of more careful prescribing.

We are unable to comment on the relative risk of specific ADRs or fatalities in relation to valproic acid use because we do not have information regarding the denominator, i.e. the number of prescriptions for valproic acid for children with epilepsy worldwide. Instead we used the overall reporting in VigiBase to generate a relative frequency for our report series. Even if we could have accessed number of the worldwide use of valproic acid in the child population, we still would not have been able to generate a reliable risk estimate using our data because of the known problem of underreporting of spontaneous reports [31], [32]. We know that valproic acid is the most frequently prescribed AED for children in several countries in Europe [33], [34] and the second most frequently prescribed AED for children in the USA, Cuba and Egypt [35]-[37]. Although the number of fatalities reported is small in relation to the number of likely prescriptions, one again needs to recognise that the vast majority of ADRs are unreported or unsuspected [31], [32]. This, unfortunately, includes serious as well as minor ADRs.

Risk factors for hepatotoxicity, pancreatitis and other serious ADRs in association with valproic acid apart from polytherapy, young age and metabolic disorders (not considered in this study) need to be further explored. Is there for example a genetic disposition in children and adults that make them more vulnerable for hepatotoxicity. Until we know more, clinicians need to be aware of the risk of rare, serious ADRs, and identify and act on early signs of hepatotoxicity [38]. They also need to recognise that polytherapy appears to be a major risk factor for many of the serious ADRs in association with valproic acid.

## Epilogue

As parents ourselves, we can understand that this paper might cause concerns in parents of children, or children themselves, using valproic acid. However, serious adverse reactions are rare, especially those leading to fatalities, and the benefit of using an antiepileptic medicine (to prevent seizures with their own risk) almost certainly outweighs the risk of serious adverse reactions that we discuss here. **If you perceive unexpected changes in your child's health, particularly those symptoms noted in the product information leaflet/package insert, we encourage you to report these to your family physician.**

## Supporting Information

Appendix S1 Caveat document: Accompanying statement to data released from the Uppsala Monitoring Centre, WHO Collaborating Centre for International Drug Monitoring.
(PDF)
