# Supplementary material for: Valproic Acid and Fatalities in Children: A Review of Individual Case Safety Reports in VigiBase
Source: PLoS One. 2014 Oct 10;9(10):e108970. doi: 10.1371/journal.pone.0108970 (PMC4193865; doi:10.1371/journal.pone.0108970)
Supplement: Appendix S1 — Caveat document: Accompanying statement to data released from the Uppsala Monitoring Centre, WHO Collaborating Centre for International Drug Monitoring. (PDF) [file pone.0108970.s001.pdf]

WHO Collaborating Centre
for International Drug Monitoring
Box 1051, SE-751 40 Uppsala, Sweden

Tel: +46-18-65 60 60
Fax: +46-18-65 60 88
E-mail: info@who-umc.org

CAVEAT DOCUMENT

Accompanying statement to data released from the Uppsala Monitoring Centre, WHO Collaborating Centre for International Drug Monitoring

Uppsala Monitoring Centre (UMC) in its role as the WHO Collaborating Centre for International Drug Monitoring receives reports of suspected adverse reactions to medicinal products from National Centres in countries participating in the WHO pharmacovigilance network, the WHO Programme for International Drug Monitoring. Limited details about each suspected adverse reaction are received by the UMC. The information is stored in the WHO Global Individual Case Safety Report database, VigiBase. It is important to understand the limitations and qualifications that apply to this information and its use.

The reports submitted to UMC generally describe no more than suspicions which have arisen from observation of an unexpected or unwanted event. In most instances it cannot be proven that a specific medicinal product (rather than, for example, underlying illness or other concomitant medication) is the cause of an event.

Reports submitted to National Centres come from both regulated and voluntary sources. Some National Centres accept reports only from medical practitioners; other National Centres accept reports from a broader range of reporters, including patients. Some National Centres include reports from pharmaceutical companies in the information submitted to UMC; other National Centres do not.

The volume of reports for a particular medicinal product may be influenced by the extent of use of the product, publicity, the nature of the reactions and other factors. No information is provided on the number of patients exposed to the product.

Some National Centres that contribute information to VigiBase make an assessment of the likelihood that a medicinal product caused the suspected reaction, while others do not.

Time from receipt of a report by a National Centre until submission to UMC varies from country to country. Information obtained from UMC may therefore differ from those obtained directly from National Centres.

For the above reasons interpretations of adverse reaction data, and particularly those based on comparisons between medicinal products, may be misleading. The supplied data come from a variety of sources. The likelihood of a causal relationship is not the same in all reports. Any use of this information must take these factors into account.

Some National Centres strongly recommend that anyone who intends to use their information should contact them for interpretation.

Any publication, in whole or in part, of information obtained from UMC must include a statement:

(i) regarding the source of the information,
(ii) that the information comes from a variety of sources, and the likelihood that the suspected adverse reaction is drug-related is not the same in all cases,
(iii) that the information does not represent the opinion of the World Health Organization.

Omission of this statement may exclude the responsible person or organization from receiving further information from VigiBase.

2011
